# Socioeconomic transitions and the spatial dynamics of syphilis transmission in urbanizing China

**DOI:** 10.1186/s12939-026-02949-1

**Published:** 2026-07-24

**Authors:** Chengcheng Li, Hanxi Zhang, Jinfeng Li, Ximan Ye, Shangcheng Zhou, Xiaojie Lao, Maowei Chen

**Affiliations:** 1 Humanities and Management School, Zhejiang Chinese Medical University, Hangzhou, Zhejiang 310001 China; 2 WHO Collaborating Centre for Comprehensive Management of HIV Treatment and Care, Beijing Ditan Hospital Capital Medical University, Beijing, 100015 China; 3 Department of Infectious Disease, Beijing Ditan Hospital Capital Medical University, NO. 8 Jingshun East Street, Chaoyang District, Beijing, 100015 China; 4 Beijing Key Laboratory of Technology and Application of Anti-Infective New Drugs Research and Development, NO. 8 Jingshun East Street, Chaoyang District, Beijing, 100015 China; 5 Infectious Diseases Department, Wuming Hospital of Guangxi Medical University, No. 26 Yongning Road, Wuming District, Nanning, Guangxi, 530199 China; 6 National Key Laboratory of Intelligent Tracking and Forecasting for Infectious Diseases, National Center for AIDS/ STD Control and Prevention, Chinese Center for Disease Control and Prevention, NO. 8 Jingshun East Street, Chaoyang District, Beijing, 102206 China

**Keywords:** Syphilis, Spatial dynamics, Economy, Education, Population mobility

## Abstract

**Background:**

Syphilis remains a re-emerging sexually transmitted infection in China, with transmission increasingly shaped by regional socioeconomic disparities. Understanding spatial heterogeneity and inter-provincial inequality is essential for equitable disease control.

**Methods:**

A balanced panel dataset of syphilis incidence was constructed across 31 provinces in mainland China from 2004 to 2020. Temporal trends were stratified by age and disease stage using joinpoint regression analysis. Spatial clustering patterns of syphilis incidence were evaluated through Global Moran’s I and Local Moran’s I statistics. To disentangle the socioeconomic determinants of spatial inequality, the Spatial Durbin Model (SDM) for regional spillovers and Multiscale Geographically Weighted Regression (MGWR) were employed to detect structural heterogeneity in risk factors associated with a regime shift external shocks (the Global Financial Crisis and COVID-19).

**Results:**

Syphilis incidence rose substantially (AAPC = 10.69%), driven predominantly by a surge in latent infections among the elderly (≥ 65 years). Spatially, the epidemic exhibited a distinct “westward shift” from affluent coastal regions to the less-developed Northwest. The SDM indicated that economic growth and population mobility generally increased risk, while educational investment exerted a robust protective spillover effect. Crucially, MGWR revealed a deepening spatial inequity in risk mechanisms: Specifically, the 2008 Financial Crisis associated with a regime shift in the less-developed Northwest, transitioning the epidemic from a “wealth-driven” model to a “poverty-driven” risk model. Furthermore, The COVID-19 pandemic inverted the role of transportation, shifting it from a protective factor associated with urban resilience into a primary vector for disease diffusion.

**Conclusions:**

Syphilis transmission in China exhibits clear spatial stratification, where the burden is increasingly shifting to resource-constrained regions. The divergence between consumption-based risk in developed areas and survival-based risk in underdeveloped hinterlands underscores how macroeconomic instability amplifies health inequalities Equity-oriented interventions must prioritize “poverty-driven” transmission pathways and vulnerable older populations to prevent the entrenchment of disease in lagging regions.

**Supplementary Information:**

The online version contains supplementary material available at https://doi.org/10.1186/s12939-026-02949-1.

## Introduction

Syphilis remains a persistent public health challenge, characterized by asymptomatic transmission and severe complications. In China, syphilis was eliminated in the 1960s through rigorous public health interventions, including mass screening, free treatment, and closure of brothels [1, 2]. However, economic policies promoting openness to the global market have profoundly altered Chinese society, setting the stage for the resurgence of syphilis and other sexually transmitted infections (STIs) [3]. Rapid economic development has increased individual wealth, enhanced regional mobility, and relaxed governmental controls, collectively fostering risky sexual behaviors and growing demand for commercial sex [4, 5]. Since the first reported case in 1979 [3], national surveillance data have shown a substantial and sustained increase in syphilis incidence over the past decades [6–9].

In 2008, with the emergency of the global financial crisis, China shifted its economic policy from an export-oriented model to domestic economic stimulation, significantly increasing infrastructure investments in high-speed railways and the internet. These measures sustained China’s economic growth, enabling it to become the world’s second-largest economy, behind only the United States. However, rapid improvements in transportation infrastructure and widening income disparities intensified migration, creating large populations of long-distance rural migrants who typically experience higher risks of sexually transmitted infections [5, 10]. Based on Global Burden of Disease Study 2019 estimates, there were approximately 242 million prevalent cases of five major STIs in China in 2019, with 173 million new incident cases reported that year, resulting in an estimated 350,000 disability-adjusted life years (DALYs) lost [11].

The period from 2004 to 2020 represents a critical window for investigating how China’s rapid socioeconomic development has reshaped the spatiotemporal dynamics of syphilis. While existing literature has documented rising syphilis incidence [6–9], empirical evidence regarding its complex spatiotemporal patterns and underlying drivers remains limited. Previous studies have frequently relied on aspatial or global regression models, which often overlook the spatial spillover effects and geographical heterogeneity inherent in infectious disease transmission.

To address these gaps, this longitudinal spatiotemporal analysis reconstructs the dynamics of syphilis and identifies its socioeconomic determinants. This study advances spatial epidemiology by integrating two complementary modeling frameworks: the Spatial Durbin Model (SDM), which quantifies global direct and spatial spillover effects of socioeconomic transitions, and Multiscale Geographically Weighted Regression (MGWR), which captures spatial non-stationarity and structural breaks triggered by external shocks (e.g., the 2008 Financial Crisis and COVID-19). Specifically, three key objectives addressed three objectives: (1) to characterize the temporal trends and demographic shifts (by age and disease stage) of the syphilis epidemic; (2) to detect spatial clustering patterns and quantify both the direct and spatial spillover effects of socioeconomic transitions; and (3) to examine the spatial non-stationarity of these associations to identify localized risk mechanisms. By transitioning from traditional aspatial approaches to an integrated spatiotemporal framework, this study provides a granular understanding of how macro-level economic transformations shape localized syphilis transmission, thereby informing more targeted and equitable public health interventions.

## Data and methods

### Study regions and data sources

A longitudinal spatiotemporal analysis was conducted to examine the epidemiological dynamics of syphilis and its socioeconomic determinants in mainland China from 2004 to 2020. The study utilized a balanced province–year panel dataset comprising 31 provincial-level administrative divisions (PLADs). Continuous national epidemiological surveillance data served as the primary basis for model construction, with the PLAD as the unit of analysis.

The 31 PLADs comprise provinces, autonomous regions, and municipalities, and Hong Kong, Macao, and Taiwan were excluded due to differing data reporting systems. These 31 PLADs were categorized into seven major regions follows the standard geographical division framework adopted by the National Bureau of Statistics of China in the ‘China Statistical Yearbook’. The classification is as follows: Northeastern China (Heilongjiang, Jilin, Liaoning), Northern China (Beijing, Tianjin, Hebei, Shanxi, Inner Mongolia), Northwestern China (Shaanxi, Gansu, Qinghai, Ningxia, Xinjiang), Eastern China (Shanghai, Jiangsu, Zhejiang, Anhui, Fujian, Jiangxi, Shandong) Central China (Henan, Hubei, Hunan), Southern China (Guangdong, Guangxi, Hainan), Southwestern China (Chongqing, Sichuan, Guizhou, Yunnan, Xizang). This classification is widely used in socioeconomic research and policy analysis in China, as it reflects both geographical continuity and regional socioeconomic characteristics [12–14]. The vector spatial data of China’s map are sourced from the Standard Map Service website of the National Geomatics Center of China (https://www.ngcc.cn/).

Syphilis outcomes were derived from China’s national mandatory notifiable disease surveillance system and compiled as annual province-level incidence rates. Publicly available mandatory report surveillance data from the Public Health Science Data Center (http://www.phsciencedata.cn/), as the primary source of case records, while data from the Chinese Center for Disease Control and Prevention (https://www.chinacdc.cn/) were used for verification and cross-checking. These routinely collected surveillance data reflect both transmission dynamics and surveillance/healthcare processes, so the analyses target population-level spatiotemporal associations rather than individual-level causal effects.

The reported cases of syphilis encompass primary syphilis, secondary syphilis, tertiary syphilis, congenital syphilis, and latent syphilis, aligning with the diagnosis criteria for syphilis (*WS 273–2018*) by the National Health Commission of the People’s Republic of China. Panel data on total syphilis incidence from 31 PLADs from 2004 to 2020 served as the dependent variable to measure syphilis transmission.

Demographic data by age and a set of seven socioeconomic indicators (detailed in Section "Socioeconomic Indicators") for the 31 PLADs from 2004 to 2020 were collected from the National Bureau of Statistics of China (https://www.stats.gov.cn/sj/pcsj/). These indicators served as explanatory variables to assess the spatial correlation and regional influence of socioeconomic development on syphilis transmission.

All data processing, integration, the creation of panel datasets, spatial visualizations and mapping were conducted using Python 3.9 with the pandas, numpy and pyechart libraries for efficient handling of large-scale data.

### Socioeconomic indicators

To assess the impact of regional socioeconomic development on syphilis transmission, seven indicators were selected from three key domains—economic level, education level, and population mobility level—based on data availability and theoretical relevance to public health outcomes (Table 1, Appendix Table S1). Economic factors included per capita GDP, unemployment rate, average wage, and the housing price ratio, reflecting regional prosperity, labor market stability, income levels, and living cost pressures, respectively. Education level was represented by per capita education expenditure, serving as a proxy for human capital investment and health literacy. Population mobility was gauged by the population growth rate and passenger-kilometers, which quantify regional migration and transportation connectivity. Detailed definitions, calculation methods, and data sources for all indicators are provided in Appendix Table S1, with all data for the 31 provincial-level administrative divisions (PLADs) during the study period (2004–2020) obtained from the National Bureau of Statistics of China (https://www.stats.gov.cn/sj/tjgb/ndtjgb/).

### Methodology

#### Joinpoint regression analysis

Temporal trends of syphilis incidence were assessed using Joinpoint regression software v5.4.0, (National Cancer Institute, Rockville, MD, USA). The default grid search method was employed to identify the optimal number and location of joinpoints, with Monte Carlo permutation tests applied to determine statistical significance. Model selection was guided by the Bayesian information criterion (BIC) to ensure goodness of fit [15]. The annual percent change (APC) was calculated for each segment to quantify the average annual percentage change in incidence rates, while the average annual percent change (AAPC) with corresponding 95% confidence intervals (CIs) was estimated as a geometrically weighted average of APCs across all intervals. An APC or AAPC > 0 with *P* < 0.05 indicated a statistically significant upward trend, whereas values < 0 with *P* < 0.05 indicated a downward trend; non-significant results (*P* ≥ 0.05) were interpreted as stable trends. The general model is expressed as Eq. (1), where $\:{y}_{t}$ is the annual incidence rate, $\:\alpha\:$ is the intercept, $\:{\beta\:}_{k}$ denotes the slope for segment * k*, $\:{\tau\:}_{k}$ is the joinpoint, and $\:{\epsilon\:}_{t}$ is the random error term. The APC and AAPC can be expressed as Eqs. (2–3), respectively, where $\:{w}_{k}$ represents the length (in years) of segment * k*.

1 $$\:\mathrm{l}\mathrm{n}\left({y}_{t}\right)=\alpha\:+{\beta\:}_{k}(t-{\tau\:}_{k})+{\varepsilon\:}_{t}$$

2 $$\:\mathrm{APC}=({e}^{{\beta\:}_{k}}-1)\times\:100{\%}$$

3 $$\:\mathrm{AAPC}=\frac{\sum_{k=1}^{m}{w}_{k}\times\:{\mathrm{APC}}_{k}}{\sum_{k=1}^{m}{w}_{k}}$$

### Global autocorrelation analysis

Spatial dependence is a common feature of infectious disease transmission. To examine whether syphilis incidence across provinces exhibited spatial clustering, Moran’s I index was applied for each year from 2004 to 2020. The formula for Global Moran’s I can be expressed as Eq. (4). *I* represents the Global Moran’s I index; where * n*is the number of spatial units, $\:{x}_{i}$ and $\:{x}_{j}$ are the observed values, $\:\stackrel{\prime }{x}\:$ is the global mean, and $\:{w}_{ij}$ is the spatial weight between regions * i* and * j*. A distance-decay spatial weight matrix was constructed using the great-circle distance between provincial capitals. The spatial weight matrix W = [w_ij_] using a distance-decay approach, in which provinces closer in distance receive higher weights, and those farther apart receive lower weights. The weighting formula can be expressed as Eq. (5). The$\:{d}_{ij}$ denotes the great-circle distance between provinces * i* and * j*, and * h* is the bandwidth parameter controlling decay rate. This formulation ensures that interactions between geographically proximate provinces are more strongly emphasized, reflecting the diminishing influence of distance on spatial relationships, particularly considering the significant scale disparity between China’s administrative divisions. The Global Moran’s I and distance-decay spatial weight matrix was calculated with GeoDa 1.20 (Luc Anselin, Urbana).

4 $$\:I=\frac{n}{{\sum}_{i}{\sum}_{j}{w}_{ij}}\cdot\:\frac{{\sum}_{i}{\sum}_{j}{w}_{ij}({x}_{i}-\bar{x})({x}_{j}-\bar{x})}{\sum_{i}({x}_{i}-\bar{x}{)}^{2}}$$

5 $$\:{w}_{ij}=\left\{\begin{array}{r}e\mathrm{xp}{\hspace{0.17em}}\left(-\frac{{d}_{ij}^{2}}{{h}^{2}}\right),\:\:i\ne\:j\\\:0,\:\:i=j\end{array}\right.$$

### Local autocorrelation analysis

To further explore the local spatial clustering characteristics of syphilis incidence, the Anselin Local Moran’s I (a type of Local Indicators of Spatial Association, LISA) was employed to identify provinces forming significant clusters or outliers in 2004 and 2020, which facilitates the assessment of spatial correlations within specific localities. This method identifies whether each spatial unit forms a statistically significant cluster or outlier by analyzing the co-variation between its value and those of its neighbors. This analysis adopted the same distance-decay-based spatial weight matrix and used Monte Carlo simulations to assess statistical significance. The formula can be expressed as Eqs. (6,7): *I*_*i*_ represents the Local Moran’s I for unit i, which measures the degree of local spatial association between the unit and its neighbors; *x*_*i*_ and *x*_*j*_: incidence in units *i* and *j*; *x *represents the overall mean incidence across all spatial units; *w*_*ij*_: represents the spatial weight between unit *i* and unit *j*; *m²*: represents the standardized variance of the variable, serving as a normalization factor for local spatial statistics. Local Moran’s I was computed using GeoDa 1.20, with Monte Carlo randomization (999 permutations) to assess significance. Visualization and analysis of the local Moran’s I results, which highlight high-high clusters, low-low clusters, and outlier provinces, were conducted using the LISA cluster map and LISA significance map within ArcGIS 10.8 (Esri, Redlands, CA, USA)

6 $$\:{I}_{i}=\frac{({x}_{i}-\bar{x})}{{m}^{2}}\sum_{j}{w}_{ij}({x}_{j}-\bar{x})$$

7 $$\:{m}^{2}=\frac{1}{n}\sum_{i}({x}_{i}-\bar{x}{)}^{2}$$

### Spatial Durbin Model (SDM)

Spatial panel models are widely used to account for spatial dependence in epidemiological data [16]. The SDM was employed to quantify the association between syphilis incidence and multiple socioeconomic factors across 31 provinces from 2004 to 2020, while allowing for spatial dependence in both the outcome and covariates from neighboring provinces. Importantly, the SDM is a global spatial econometric model and therefore yields spatially homogeneous (average) effects over the entire study area. The SDM can be expressed as Eq. (8): $\:\mathrm{y}$= vector of dependent variables (syphilis incidence); $\:\mathrm{X}$= matrix of independent variables; $\:\mathrm{W}$= spatial weight matrix; $\:\rho\:$= spatial autoregressive coefficient; $\:\beta\:$ and $\:\theta\:$= coefficients for local and spillover effects; $\:\epsilon\:$= error term. The SDM was estimated using Stata 15.0 (StataCorp, College Station, TX, USA). The Hausman and likelihood ratio (LR) tests were conducted to determine the appropriateness of fixed effects, and results supported the use of a fixed effects SDM. Additionally, LR and Wald tests were used to verify whether the SDM could be reduced to the Spatial Lag Model (SLM) or Spatial Error Model (SEM). Monte Carlo simulations were performed to assess robustness. A *P* value *<* 0.05 is generally considered to indicate statistical significance.

8 $$\:\mathrm{y}=\rho\:\mathrm{W}\mathrm{y}+\mathrm{X}\beta\:+\mathrm{W}\mathrm{X}\theta\:+\varepsilon\:$$

Given that the analysis focused on PLADs, a fixed-effects specification was employed. It is important to note that the SDM estimates represent average spatial associations rather than direct causal effects. Since the regression coefficients in the Spatial Durbin Model (SDM) cannot be directly interpreted as marginal effects, a partial differentiation approach was employed to decompose the total effects into the average direct effect (ADE) (within-province impact, averaged over provinces), and the average indirect effect (AIE) (cross-province spillover impact, averaged over provinces). These ADE/AIE estimates should be interpreted as global average effects across PLADs under the assumed spatial interaction structure.

### Multiscale Geographically Weighted Regression (MGWR)

To examine whether the associations identified by the global SDM vary across space (i.e., spatial non-stationarity), MGWR was implemented as a complementary local modeling approach [17]. MGWR serves as an exploratory diagnostic tool for spatial heterogeneity, allowing regression parameters to vary geographically and permitting each explanatory variable to operate at its own spatial scale via variable-specific bandwidths. It should be emphasized that MGWR results are exploratory in nature, intended to identify localized patterns and diagnostic insights into spatial variations rather than to establish causal relationships. The general form of the MGWR model is expressed as Eq. (9): where $\:b{w}_{k}$ denotes the optimal bandwidth specific to variable $\:{x}_{k}$. This enables simultaneous modeling of both local and regional processes, providing a more realistic representation of spatial heterogeneity. The MGWR analysis was implemented using MGWR Version 2.2.1, developed by the Spatial Analysis Research Center (SPARC) at Arizona State University [18].

9 $$\begin{gathered} \:{y_i} = \beta {\:_0}({u_i},{v_i}) \hfill \\ + \sum {\:_{k = 1}^p} \beta {\:_k}(b{w_k};{u_i},{v_i}){x_{ik}} + {\varepsilon _i} \hfill \\ \end{gathered}$$

Finally, the spatial distributions of local parameter estimates were mapped to identify where socioeconomic determinants exhibited stronger/weaker associations with syphilis incidence, thereby complementing the SDM results and informing interpretation under potential spatial heterogeneity.

## Results

### Overview of syphilis epidemiology

**Fig. 1:**
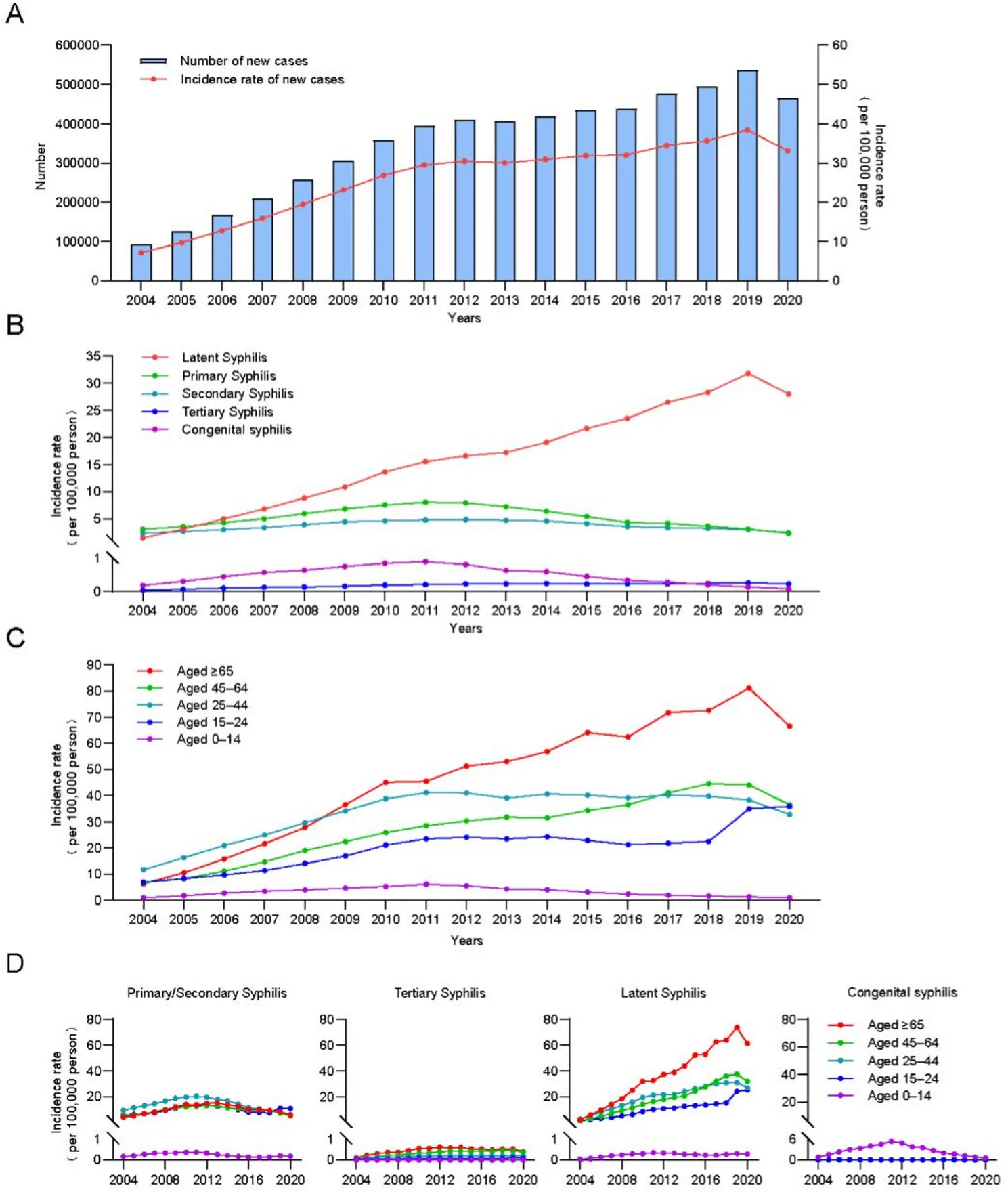
Temporal trends of syphilis incidence in China, 2004–2020. **A** Annual number and incidence rate of newly reported syphilis cases, 2004–2020. **B** Incidence rate trends of syphilis by stage, 2004–2020. **C** Number of new syphilis cases by age group, 2004–2020. **D** Incidence rate of syphilis by age group, 2004–2020

From 2004 to 2020, syphilis incidence in China rose substantially (AAPC = 10.69%, 95% CI: 9.83–11.55%), rising from 92,573 (7.12/100,000) in 2004 to 464,435 (33.08/100,000) in 2020 (Fig. 1A). This trajectory was primarily driven by the rapid expansion of latent syphilis (AAPC = 23.40%, 95% CI: 19.68–27.23), while primary, secondary, and congenital syphilis exhibited stable or downward trends (Fig. 1B). Overall, the escalating disease burden is overwhelmingly attributed to the surge in subclinical latent infections.

### Temporal trends of syphilis

Syphilis burden shifted progressively toward older populations over the study period (Fig. 1C). Adults aged ≥ 65 years experienced the most significant increase (AAPC = 16.35%, 95% CI: 13.59–19.17%), followed by the 45–64 age group (AAPC = 10.39%, 95% CI: 8.02–12.81). These age-specific patterns indicate a progressive aging of the syphilis epidemic and provide the basis for further examining how disease stages evolve across different age strata. Stratified analysis revealed a clear stage–age divergence (Fig. 1D): latent syphilis incidence escalated monotonically with age, peaking in the ≥ 65 age group (AAPC = 24.89%, 95% CI: 19.05–31.01). Conversely, primary and secondary syphilis were concentrated among the 25–44 age group, showing a significant downward trend (AAPC = − 2.69%, 95% CI: − 3.84 to − 1.53). Tertiary syphilis, though low in magnitude, displayed significant positive temporal slopes in older adults—most notably in 45–64 years (AAPC + 14.57%, 95% CI: 12.28–16.92) and ≥ 65 years (+ 11.61%, 95% CI: 6.44–17.02). Congenital syphilis continued to decline (AAPC − 3.44%, 95% CI: − 5.98 to − 0.83), confirming the effectiveness of maternal health interventions. These results indicate a structural aging of the syphilis epidemic.

### Spatiotemporal distributions of syphilis

**Fig. 2:**
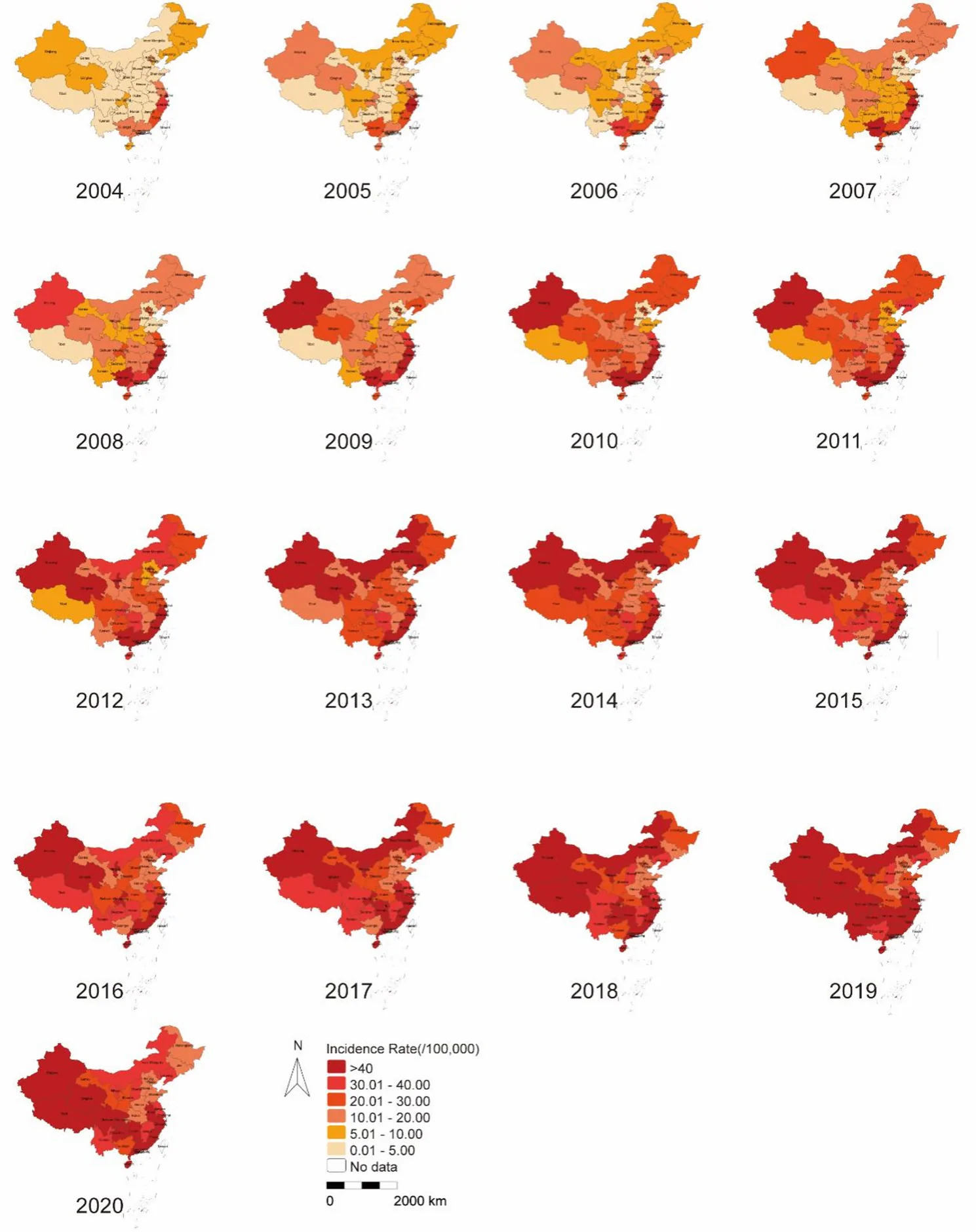
Provincial-level Spatiotemporal distribution of syphilis incidence in mainland China, 2004–2020. Annual provincial-level incidence rates (/100,000 population) are shown using a sequential color gradient, illustrating the temporal and geographical evolution of syphilis burden

The spatiotemporal evolution of syphilis incidence demonstrated pronounced geographic redistribution over the study period (Fig. 2). In 2004, the highest incidence rates were observed in Shanghai (54.64/100,000) Zhejiang (30.96/100,000) and Fujian (22.12/100,000), whereas Tibet (0.19/100,000), Hebei (0.82/100,000), and Yunnan (1.55/100,000) reported the lowest levels. By 2020, the pattern had shifted markedly toward the western and southern regions. Qinghai (74.87/100,000) emerged as the province with the highest incidence, followed by Hainan (70.08/100,000) and Xinjiang (67.12/100,000), reflecting a clear westward and southward intensification of the epidemic. In contrast, several historically low-incidence northeastern provinces, such as Hebei, Jilin and Heilongjiang, also exceeded 10.00/100,000 by 2020, indicating a rising disease burden even in previously low-risk regions.

### Spatiotemporal clustering of syphilis

Spatial autocorrelation analysis revealed a “de-clustering to re-clustering” transition.

**Fig. 3:**
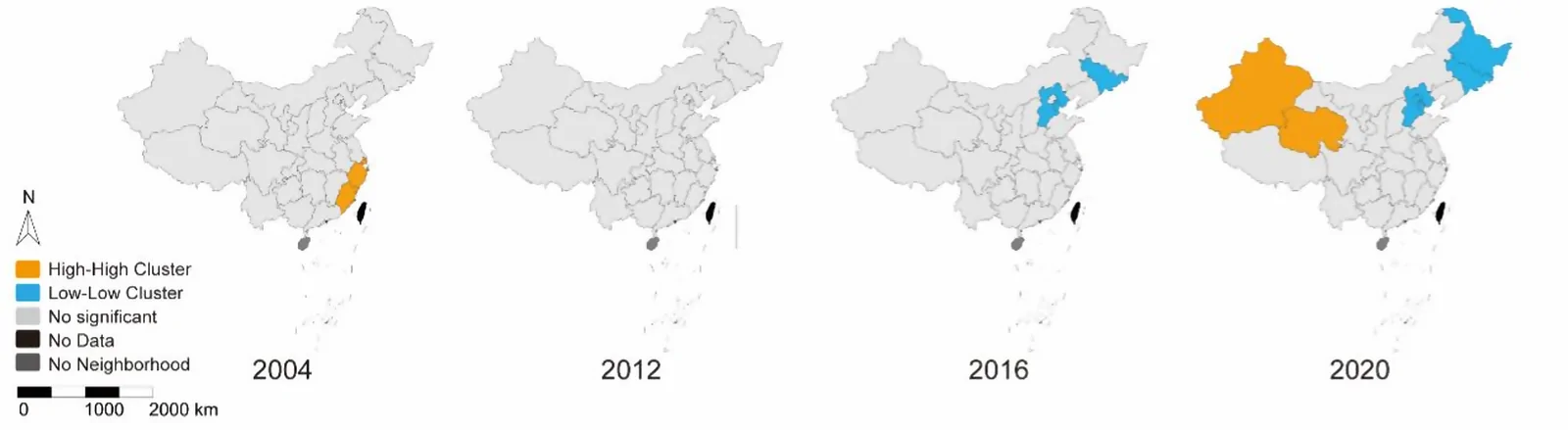
Spatial clustering distribution of syphilis incidence in China form 2004 to 2020. High-incidence clusters of syphiliswere concentrated in the eastern areas in 2004 and shifted to the southwest areas in 2020. Most areas showed no significant clustering; Hainan had no neighborhood, and regions like Taiwan had no data

Global Moran’s I analysis revealed three distinct clustering phases: a decline toward randomness (2004–2013), followed by a reemergence of spatial clustering (2014–2020) (Appendix Figure S1). LISA cluster maps corroborated these temporal shifts (Fig. 3). Significant high-incidence hotspots (High-High clusters) shifted from the southeastern coastal provinces (2004–2011) to the Northwest (Qinghai and Xinjiang, 2016–2020). Meanwhile, cold spots (Low-Low clusters) became established across parts of Northern and Northeastern China in the latter phase, such as Hebei, Jilin, Tianjin, Heilongjiang, and Beijing, illustrating a clear divergence between expanding high-risk clusters and stabilized low-risk regions. The spatial patterns identified by the Local Moran’s I analysis were consistent with the global spatial autocorrelation trends (Appendix Table S2).

### Socioeconomic drivers of syphilis transmission

Descriptive statistics for the study variables are summarized in Table 1. All indicators showed regional variation. SDM results showed GDP per capita, unemployment, education expenditure, and passenger volume and turnover had significant effects (*P* < 0.05). When spatial lags were included, average wage and housing price ratio also became significant (*P* < 0.05), while population growth remained non-significant (*P* > 0.05) (Appendix Table 3).

Hausman test (χ² = 23.37, *P* < 0.01) and Lagrange Multiplier (LM) test (χ²=689.13, *P* < 0.001) supported a fixed-effects model. LR test rejected simplifying the SDM to Spatial Lag Model (SLM) (χ² = 31.40, *P* < 0.01) or Spatial Error Model (SEM) (χ² = 142.79, *P* < 0.01), supporting SDM as optimal. Due to spatial lag effects, regression coefficients were decomposed into direct and indirect (spillover) effects using partial derivatives.

The SDM results indicated that economic development and population mobility were primary drivers of syphilis incidence (Table 2). Specifically, per capita GDP, unemployment rate, and passenger-kilometers as significant direct contributors to syphilis incidence (ADE = 16.51, 6.46, 16.62; *P* < 0.05), highlighting the roles of economic activity and population mobility. Indirect effect (spillover effects) revealed unemployment rate in neighboring provinces showed a protective influence (AIE=-21.60, *P* < 0.001), while higher average wages and housing price ratios in nearby provinces increased local syphilis risk (AIE = 25.54 and 228.21; *P* < 0.05). Additionally, education expenditure had significant negative direct and indirect effects (ADE = -11.22 and AIE = -25.62; *P* < 0.05). No significant effect was observed for population growth.

**Table 1:** Summary statistics of variables

Variable	Symbol	Mean	SD	Min	Max
Syphilis incidence (per 100,000)	lin	31.35	19.49	1.41	107.51
Per capita GDP (log)	lngdp	10.56	0.58	9.20	11.78
Unemployment rate	ur	3.42	0.66	1.21	4.60
Average wage (log)	lnaw	10.73	0.37	9.95	11.79
Housing price ratio	hr	0.12	0.42	0.04	0.28
Per capita education expenditure (log)	lnpmu	5.51	0.50	3.904	6.54
Population growth	np	28.76	66.30	-319	8.03
Passenger-kilometers (log)	lnpt	6.29	0.94	3.40	8.01

**Table 2:** Direct and spillover effects from spatial durbin model

Variable	ADE	*P*	AIE	*P*
Per capita GDP (log)	16.51	0.021	-0.50	0.962
Unemployment rate	6.46	0.001	-21.60	< 0.001
Average wage (log)	-6.61	0.420	25.54	0.006
Housing price ratio	-39.89	0.318	228.21	0.005
Per capita education expenditure (log)	-11.22	0.035	-25.65	0.032
Population growth	0.00	0.723	0.03	0.299
Passenger-kilometers (log)	16.62	< 0.001	8.02	0.403

ADE, Average Direct Effect; AIE, Average Indirect Effect

### Structural shifts in socioeconomic driving mechanisms

During the study period (2004–2020), Chinese society experienced two external structural shifts: the Global Financial Crisis and the COVID-19 pandemic. Given that the Spatial Durbin Model (SDM) assumes spatial homogeneity and yields only global average estimates, MGWR was employed to investigate spatial heterogeneity and investigate whether the spatial associations exhibited structural variations were associated with these specific periods. The results revealed significant non-stationarity in driving factors (Table 3).

**Fig. 4:**
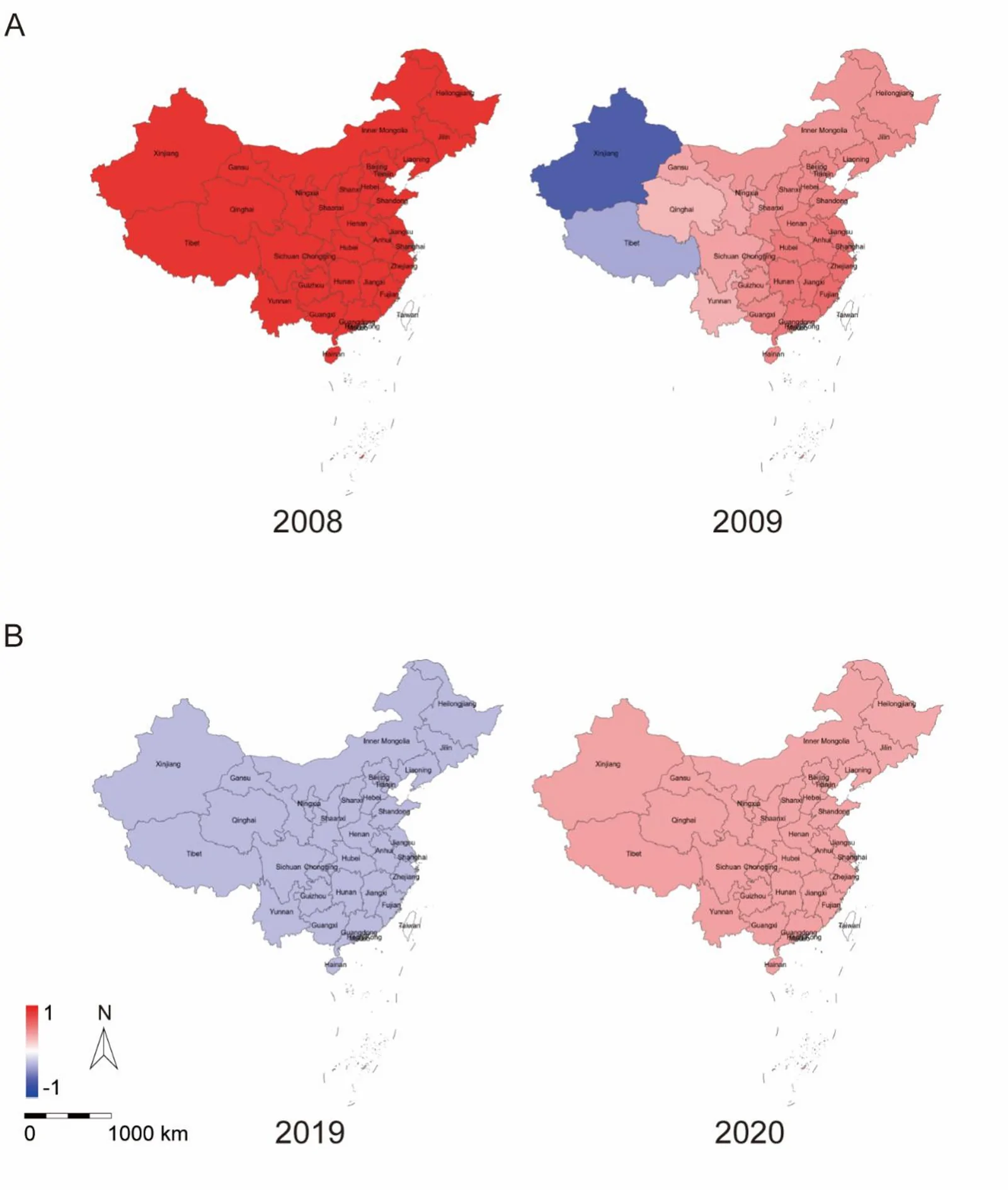
Structural shifts in syphilis determinants associated with the global financial crisis and the COVID-19 Pandemic. **(A)** Spatial disparity of Economic Gradients (2008–2009): The impact of average wage shifted from a globally consistent positive driver in 2008 (“wealth-driven” pattern) to a spatially polarized mechanism in 2009. The emergence of negative coefficients (blue) in Northwest China indicates a transition to a “poverty-driven” risk model during the post-crisis recession. **(B) **Directional Inversion of Transport Mobility Vectors (2019–2020): The relationship between passenger transport and incidence flipped completely. It transitioned from a negative predictor in 2019 (indicating the protective resilience of developed urban hubs) to a positive risk factor in 2020 (red), suggesting that the pandemic stripped away urban medical advantages and restored transport’s primary role as a transmission vector

Regarding the impact of the Global Financial Crisis (2008–2009), the MGWR results indicated notable changes in the model fit and the spatial scale of the wage variable. In 2008, the average wage was a positive predictor with a mean coefficient of 0.822, operating at a global scale with a bandwidth of 78.11. However, in 2009, the mean coefficient decreased to 0.383, and the bandwidth reduced to 9.70, indicating a shift from a global to a localized spatial disparity process. The effect values weakened sequentially from the Southeast region to the Northwest, with the regions of Tibet and Xinjiang ultimately exhibiting negative coefficients (Fig. 4A). Concurrently, the explanatory power of the model decreased, with the adjusted R^2^ dropping from 0.844 in 2008 to 0.574 in 2009.

Regarding the impact of the COVID-19 pandemic (2019–2020), the analysis revealed a directional change in the relationship between passenger transport and syphilis incidence. In 2019, passenger transport showed a negative association with incidence (Mean Coeff = -0.247) and operated at a global bandwidth of 78.11. In 2020, this coefficient shifted to a positive value (Mean Coeff = 0.337), and the bandwidth decreased to 33.87, reflecting a directional Inversion in spatial effect (Fig. 4B). In contrast to the 2008–2009 period, the overall model fit remained stable, with the adjusted R^2^ increasing slightly from 0.668 in 2019 to 0.686 in 2020.

**Table 3:** Structural breaks in syphilis determinants: comparison of MGWR parameters during the global financial crisis and COVID-19 pandemic

Period Shock	Variable	Metric	Shock Year	
**A. Global Financial Crisis**			2008 (Crisis)	2009 (Post- Crisis)
	Average wage	Mean Coeff	0.822	0.383
		Bandwidth	78.110	9.700
		Adj. t-val (95%)	2.044	2.387
	Model Fit	*Adj. R* ^*2*^	*0.844*	*0.574*
**B. COVID-19 Pandemic**			**2019 (Pre COVID)**	**2020 (COVID)**
	Passenger-kilometers	Mean Coeff	-0.247	0.337
		Bandwidth	78.110	33.870
		Adj. t-val (95%)	2.046	2.063
	Model Fit	*Adj. R^* ^*2*^	*0.668*	*0.686*

1. Period Shock: the fragmentation of the wage-risk relationship during the Financial Crisis (2008–2009) and the directional reversal of transportation impact during the COVID-19 pandemic (2019–2020)

2. Bandwidth: A bandwidth of ~ 78 indicates a global process (stationary), while smaller bandwidths indicate localized spatial processes (heterogeneity)

3. Model Specification: All coefficients and R² values are derived from the MGWR model, adjusting for GDP, unemployment, housing, education, and population growth

## Discussion

This nationwide study demonstrates substantial spatiotemporal heterogeneity in syphilis incidence across China, with a persistent rise in high-burden regions. By integrating surveillance data with socioeconomic indicators, it highlights the influence of economic development, education, and population mobility on syphilis transmission. Crucially, the application of MGWR further reveals that these driving mechanisms are not static; rather, they exhibit significant spatial non-stationarity and are profoundly exhibit variations by major external shocks such as the Global Financial Crisis and the COVID-19 pandemic. These findings underscore the importance of addressing structural determinants in guiding targeted prevention and control strategies.

The epidemiological profile of syphilis in China is undergoing a structural transition characterized by a stage-age divergence. The rapid escalation of latent syphilis, particularly among older populations, indicates an aging-related vulnerability and enhanced case detection efficiency [19–21] This shift suggests that the primary transmission reservoir has migrated from younger, sexually active cohorts toward middle-aged and elderly groups, a transition likely facilitated by changing social networks and evolving health-seeking behaviors. In contrast, the sustained decline in congenital syphilis serves as a benchmark for the success of targeted maternal health interventions, confirming that systemic clinical service improvements effectively interrupt specific transmission pathways [22–25]. The interplay between the increasing latent burden in older adults and the contraction of vertically transmitted infections reveals a fragmented epidemic landscape where diverse socioeconomic and healthcare resource disparities dictate the regional impact and control efficacy [26, 27].

The spatiotemporal evolution of syphilis in China signals a fundamental shift in disease transmission dynamics, moving from a concentration in affluent eastern coastal provinces to a more decentralized, high-burden profile in the Northwest. These results consistent with previous studies [28, 29] and suggests that the epidemic has moved beyond its traditional urban hubs. The geographic expansion of the syphilis burden is closely tied to the internal migration patterns and the uneven economic landscape that has reshaped China’s inland provinces over the past two decades Ultimately, these trends highlight a strong association between syphilis transmission and the broader economic transitions currently unfolding across China.

The socioeconomic determinants of syphilis in China reveal a complex, dual-directional relationship with economic development. Both higher per capita GDP and rising unemployment rates were significantly associated with increased local syphilis incidence. Prior studies showed conflicting findings—syphilis is often more prevalent in poor regions of high-income countries, such as the US [30, 31]. However, higher in affluent areas of low- and middle-income countries, including China [32]. These results support a U-shaped relationship between economic prosperity and syphilis risk: while rapid growth often facilitates infection through increased population mobility and shifting social norms [33], economic downturn, marked by high unemployment, exacerbate social vulnerability and hinder healthcare access [34–36]. Furthermore, the observed spatial spillover effects of housing prices and wages suggest that local disease burdens are deeply embedded in regional labor markets, where rising living costs act as a catalyst for migration-induced disease diffusion. Collectively, this interplay underscores that syphilis risk in China is not merely a product of poverty or affluence, but a dynamic byproduct of the socioeconomic transition.

Large-scale internal migration serves as a primary vector for the spatial propagation of syphilis in China. The observed correlation between passenger-kilometers and syphilis incidence highlights how modern transportation networks facilitate the cross-regional spread of the pathogen. According to the 2016 China Migrant Population Development Report, the internal migrant population exceeded 200 million since 2004, with a declining share in the southeast and growing migration into northwestern provinces [37]. This demographic trend parallels the spatial expansion of syphilis from affluent eastern provinces to inland regions, suggesting that infrastructure-driven mobility has reshaped the national epidemic landscape [38]. This mobility-driven transmission is particularly pronounced among high-risk groups [39]. Rural-to-urban migrants are significantly more likely to contract sexually transmitted infection (STIs) compared to the general population [40]; Previous studies have documented syphilis prevalence rates as high as 46.8% among migrant workers, alongside rapid annual increases reaching 20.72% among university students [41]. These demographic vulnerabilities, combined with the scale of human movement, disrupt established social and medical support networks and increase the likelihood of high-risk sexual encounters. Consequently, the spatial expansion of syphilis from coastal hubs toward the Northwest reflects how national infrastructure development has inadvertently increased the permeability of regional boundaries to infectious diseases.

Education emerges as a critical protective effect against syphilis transmission. Increased per capita education spending was significantly associated with lower syphilis incidence locally and regionally. Education may enhance health literacy, sexual health awareness, and protective behaviors [42]. Previous studies link low educational attainment to higher syphilis prevalence, likely due to reduced knowledge and limited access to services [22]. The observed regional spillover effect suggests that education can promote positive externalities through social interaction and migration, supporting the case for integrated regional health strategies. Increased investment in health education, particularly targeting mobile and high-risk populations, is essential [2, 43].

Beyond global average effects, the MGWR analysis uncovers critical structural breaks in these driving mechanisms associated with external shocks. First, the Global Financial Crisis (2008–2009) associated with a fragmentation in the wage-risk relationship that provides a mechanistic explanation for the observed westward shift of the epidemic. While higher wages were a consistent risk factor in 2008 (reflecting the “wealth-driven” nature of commercial sex consumption), the post-crisis period in 2009 saw the emergence of negative coefficients in the less-developed Northwest specifically in the less-developed Northwest. This implies that the spatial relocation of high-incidence clusters toward the northwest was observed alongside a distinct regime shift; a transition toward a “poverty-driven” risk model [30]. In these economically fragile regions, unlike the discretionary consumption type in coastal areas, economic hardship likely compelled vulnerable populations into survival-type, thereby establishing the Northwest as a new, poverty-associated reservoir of infection [44]. Second, the COVID-19 pandemic (2019–2020) was associated with a directional inversion in the role of transportation. In 2019, passenger transport was negatively associated with incidence, likely because transport hubs correlate with better urban medical resources and screening capacity. However, in 2020, this relationship flipped to positive, indicating that during a pandemic, the transport network reverted to its primary function as a vector for disease diffusion, overriding the protective benefits of urban infrastructure [45, 46]. These shifts demonstrate that the drivers of syphilis are highly sensitive to macroeconomic and social stability.

The identified spatiotemporal dynamics and socioeconomic drivers provide three actionable directives for updating China’s syphilis control strategy. First, responding to the demographic shift: the surge in latent syphilis among older adults (≥ 65 years) indicates that traditional interventions targeting young, high-risk populations are no longer insufficient. It is recommended to integrate syphilis screening into the existing National Basic Public Health Services for the elderly, specifically leveraging routine visits for chronic diseases (e.g., hypertension and diabetes) to facilitate the detection of occult infections. Second, addressing spatial spillovers and regional disparities: for economically developed coastal areas, interventions should focus on health education for high-mobility populations and modernizing diagnostic infrastructure. Conversely, for economically lagging northwestern areas, the transition toward a “poverty-driven” transmission model necessitates a poverty-reduction-aligned approach, prioritizing social support and low-threshold screening services to break the cycle between economic hardship and infection. Third, adapting to structural shocks: the non-stationarity revealed by MGWR suggests that risk factors are not static. Public health surveillance systems should evolve to be “shock-responsive,” incorporating real-time socioeconomic indicators (e.g., migration shifts during economic downturns or pandemics) to dynamically allocate resources during periods of macroeconomic instability [47].

Several limitations are outlined below. First, the analysis was conducted at the provincial level, which may mask substantial within-province heterogeneity in syphilis risk and its determinants. Future work should consider higher-resolution units (e.g., prefecture/county level or gridded products) where data availability allows. Second, the set of explanatory variables was constrained by data accessibility and comparability over the study period. Important contextual factors—such as climatic and environmental conditions—were not fully incorporated, which may lead to omitted-variable bias. Third, the COVID-19 pandemic likely introduced structural breaks in both syphilis transmission dynamics and surveillance processes. The MGWR-based comparison between 2019 and 2020 provides an exploratory assessment of shock-related spatial heterogeneity. Future studies should model the pandemic period explicitly using dedicated designs (e.g., interrupted time-series, segmented/regime-specific models). Finally, it is essential to acknowledge that the identification of these structural variations does not confirm a direct causal effect of the shocks themselves. Other unobserved socioeconomic factors or simultaneous policy changes may have contributed to these observed shifts, and thus these findings should be viewed as evidence associated with broad structural transitions rather than direct evidence of causality.

## Conclusion

These results highlight a distinct westward shift in syphilis burden and a surge in latent infections among the elderly in China (2004–2020). Spatial modeling confirms that transmission is driven by the interplay of economic growth and migration spillovers, with education acting as a protective buffer. Crucially, MGWR analysis reveals that these driving mechanisms are structurally sensitive to external shocks: the Global Financial Crisis was associated with a transition from a “wealth-driven” to a “poverty-driven” risk model in the Northwest, while COVID-19 was linked with a shift the influence of transportation from a protective factor to a primary diffusion vector. Consequently, future control strategies must be “shock-responsive,” prioritizing aging populations and adapting dynamically to macroeconomic instability to prevent regional resurgence.

## Supplementary Information

Below is the link to the electronic supplementary material.

Supplementary Material 1

## Data Availability

The data and code used to generate the results in this study are available from the authors upon reasonable request.

## Abbreviations

AAPC: Average annual percent change
ADE: Average Direct Effect
AIE: Average Indirect Effect
APC: Annual percent change
CIs: Confidence intervals
GDP: Gross Domestic Product
GIS: Geographic Information Systems
LM: Lagrange Multiplier
LR: Likelihood Ratio
MGWR: Multiscale Geographically Weighted Regression
PLADs: Provincial-level administrative divisions
SEM: Spatial Error Model
SDGs: Sustainable Development Goals
SDM: Spatial Durbin Model
SLM: Spatial Lag Model
STI: Sexually Transmitted Infection
