# Supplementary material for: Socioeconomic transitions and the spatial dynamics of syphilis transmission in urbanizing China
Source: Int J Equity Health. 2026 Jul 24;25:247. doi: 10.1186/s12939-026-02949-1 (PMC13682149; doi:10.1186/s12939-026-02949-1)
Supplement: Supplementary file 1 [file 12939_2026_2949_MOESM1_ESM.docx]

**Supplemental material**

**Appendix Figure S1. Spatial clustering distribution of syphilis incidence in China from 2004 to 2020**

Scatterplots show positive slopes and increasing Moran’s I values, indicating intensifying spatial clustering across provinces from 2004 to 2020. The value of Moran’s I ranges from -1 to 1: I > 0 (significant): indicates positive spatial autocorrelation (i.e., clustering of high or low values); I < 0 (significant): indicates negative spatial autocorrelation (i.e., dispersed distribution of high and low values); I ≈ 0 (non-significant): indicates no spatial correlation (random distribution).

**Appendix Table S1: Description of Socioeconomic Indicators**

| **Domain** | **Indicator** | **Measurement** | **Data Source & Calculation** | **Theoretical Relevance** |
| --- | --- | --- | --- | --- |
| Economic Level | Per Capita GDP | Regional gross domestic product per capita. | National Bureau of Statistics;  Calculated as: Regional annual GDP / Annual average population. | Measures overall economic development & public health funding capacity |
|  | Unemployment Rate | % of unemployed individuals in the labor force. | National Bureau of Statistics; | Labor market health; high rates may correlate with vulnerability |
|  | Average Wage | Average annual wage of employed individuals. | National Bureau of Statistics; | Proxies individual income level and ability to afford health services |
|  | Housing Price Ratio | Ratio of average housing price to average annual income. | National Bureau of Statistics;  Calculated as: Average urban housing price / Average annual wage. | Measures living cost pressure, potentially crowding out health expenditures. |
| Education Level | Per Capita Education Expenditure | Government expenditure on education per capita. | National Bureau of Statistics;  Calculated as: Total government education expenditure /total population. | Reflects public investment in human capital & health literacy |
| Population Mobility Level | Population Growth Rate | Annual % change in population. | National Bureau of Statistics;  Calculated as: (Populationₜ - Population₋₁) / Population₋₁ × 100%. | Captures net migration (mechanical growth), a key driver of infection influx/export. |
|  | Passenger-Kilometers | Total volume of passenger transport (persons × distance). | National Bureau of Statistics; Calculated as: Sum across all transportation modes (rail, road, water, air). | Directly measures the intensity of population movement and connectivity |

**Appendix Table S2: Local Indicators of Spatial Association (LISA) Results for Syphilis Incidence in China (2008–2020)**

| **Year** | **Hot Spots (High-High Clusters)** | **Cold Spots (Low-Low Clusters)** |
| --- | --- | --- |
| 2008 | Fujian, Shanghai, Zhejiang | — |
| 2009 | Fujian, Shanghai, Zhejiang | — |
| 2010 | Fujian, Shanghai, Zhejiang | — |
| 2011 | Fujian, Shanghai, Zhejiang | — |
| 2012 | — | — |
| 2013 | — | — |
| 2014 | — | — |
| 2015 | — | — |
| 2016 | — | Hebei, Jilin |
| 2017 | — | Hebei, Jilin |
| 2018 | Qinghai, Xinjiang | Hebei, Heilongjiang, Jilin, Tianjin |
| 2019 | Xinjiang | Hebei, Heilongjiang, Jilin, Shandong, Tianjin |
| 2020 | Qinghai, Xinjiang | Beijing, Hebei, Heilongjiang, Jilin, Tianjin |

This table lists provinces identified as statistically significant ( *p*< 0.05) hot spots (High-High clusters) and cold spots (Low-Low clusters) based on Local Moran's I analysis. A dash ("—") indicates no significant spatial cluster was detected in that year.

**Appendix Table S3. Estimation Results of the Spatial Durbin Model**

| **Variable** | **Coefficient** | **SE** | **P** | **Spatial lag (Wx*)** | **Coefficient** | **SE** | **P** |
| --- | --- | --- | --- | --- | --- | --- | --- |
| Per capita GDP (log) | 16.82 | 7.43 | 0.024 | Wx*GDP | -2.3 | 10.36 | 0.825 |
| Unemployment rate | 6.54 | 1.94 | 0.001 | Wx*Unemployment | -21.41 | 4.72 | <0.001 |
| Average wage (log) | -7.34 | 8.66 | 0.397 | Wx*Wage | 25.67 | 9.57 | 0.007 |
| Housing price ratio | -45.05 | 40.4 | 0.265 | Wx*Housing price | 231.56 | 79.29 | 0.003 |
| Per capita education expenditure (log) | -10.79 | 5.19 | 0.038 | Wx*Education | -23.88 | 10.89 | 0.028 |
| Population growth | 0.002 | 0.008 | 0.813 | Wx*Population | 0.02 | 0.02 | 0.272 |
| Passenger-kilometers (log) | 16.59 | 4.26 | <0.001 | Wx*Passenger | 7.17 | 8.98 | 0.424 |
